# Coordinate-based simulation of pair distance distribution functions for small and large molecular assemblies: implementation and applications

**DOI:** 10.1107/S1600576724007222

**Published:** 2024-09-17

**Authors:** Xiaobing Zuo, David M. Tiede

**Affiliations:** a X-ray Science Division Argonne National Laboratory Lemont IllinoisUSA; b Chemical Sciences and Engineering Division Argonne National Laboratory Lemont IllinoisUSA; Universität Duisburg-Essen, Germany

**Keywords:** X-ray scattering, pair distance distribution functions, coordinate-based simulation, molecular assemblies, nanoscale materials, *SolX 3.0*

## Abstract

X-ray scattering has become a major tool in the structural characterization of nanoscale materials. Thanks to the widely available experimental and computational atomic models, coordinate-based X-ray scattering simulation has played a crucial role in data interpretation in the past two decades. However, simulation of real-space pair distance distribution functions (PDDFs) from small- and wide-angle X-ray scattering, SAXS/WAXS, has been relatively less exploited. This study presents a comparison of PDDF simulation methods, which are applied to molecular structures that range in size from β-cyclo­dextrin [1 kDa molecular weight (MW), 66 non-hydrogen atoms] to the satellite tobacco mosaic virus capsid (1.1 MDa MW, 81 960 non-hydrogen atoms). The results demonstrate the power of interpretation of experimental SAXS/WAXS from the real-space view, particularly by providing a more intuitive method for understanding of partial structure contributions. Furthermore, the computational efficiency of PDDF simulation algorithms makes them attractive as approaches for the analysis of large nanoscale materials and biological assemblies. The simulation methods demonstrated in this article have been implemented in stand-alone software, *SolX 3.0*, which is available to download from https://12idb.xray.aps.anl.gov/solx.html.

## Abstract 2

Methods for calculation of pair distance distribution functions from coordinate models are evaluated in terms of trade-offs between computation time and accuracy. The results demonstrate approaches that are well suited for application to large nanoscale materials and biological assemblies (>1 MDa molecular weight, >10^5^ non-hydrogen atoms).

## 1. Introduction

Owing to the advances in synchrotron techniques and state-of-the-art area X-ray detectors, synchrotron-based X-ray scattering (XS) has been widely applied for structural characterization of solution-state molecular and nanoscale assemblies because the scattering techniques cover a wide range of length scales and can be applied *in situ* and under *operando* conditions. Interpretation of small-angle (SAXS) and wide-angle (WAXS) XS has been greatly aided by the development of computational approaches that allow experimental XS patterns to be compared with scattering calculated from model structures (Svergun *et al.*, 1995 ▸; Zhang *et al.*, 2000 ▸; Zuo *et al.*, 2006 ▸; Grishaev *et al.*, 2010 ▸; Schneidman-Duhovny *et al.*, 2010 ▸, 2012 ▸; Poitevin *et al.*, 2011 ▸; Liu *et al.*, 2012 ▸; Grudinin *et al.*, 2017 ▸; Knight & Hub, 2015 ▸; Putnam *et al.*, 2007 ▸; Graewert & Svergun, 2013 ▸; Brosey & Tainer, 2019 ▸). Even though simulation of reciprocal-space scattering provides opportunities for quantitative model verification and data interpretation, the development of companion analysis in real space is of interest as it provides a more intuitive approach. Real-space structural analysis from atom-pair distribution functions is routinely performed from high-energy XS data (Billinge & Kanatzidis, 2004 ▸; Terban & Billinge, 2022 ▸), but model structure testing using pair distance distribution functions, PDDFs, obtained from lower-resolution SAXS/WAXS data has been less widely utilized.

In current practice, PDDFs from experimental SAXS/WAXS data are typically obtained through the indirect Fourier transform (IFT) because of the limited reciprocal-space data range. In the IFT method, the PDDF profile is initially guessed, converted to its XS counterpart, and then compared with scattering obtained from either experiment or simulation. The PDDF profile is iteratively modified with various regulation methods to achieve the best fit to the target XS data (Svergun, 1992 ▸; Bergmann *et al.*, 2000 ▸; Hansen, 2000 ▸; Moore, 1980 ▸). Computer programs that utilize the IFT are available, including *GNOM* (Svergun, 1992 ▸), *BayesApp* (Hansen, 2012 ▸) and *RAW* (Hopkins *et al.*, 2017 ▸). However, there are a couple of general problems of the IFT method. First, the solution of the PDDF is not unique, being potentially dependent on the restraints imposed in the fitting process. Second, artefacts could be introduced into the resulting PDDFs: for example, false oscillations, loss of peaks due to over-smoothing, and ambiguities in maximum distance, Dmax, or PDDF long-distance tails due to different data selection or software operations. Algorithms for calculation of SAXS/WAXS have been developed that tabulate PDDFs from coordinate models (Schneidman-Duhovny *et al.*, 2013 ▸) or from radial distribution functions extracted from molecular dynamics (MD) simulations (Dohn *et al.*, 2015 ▸), which are then numerically transformed to yield SAXS/WAXS for comparison with experimental data. Notably, these algorithms demonstrate the computational efficiency of calculating SAXS/WAXS from simulated PDDFs, but the experiment and models are not compared using the real-space profiles.

Here, we present a comparison of three computational methods for generating corresponding pairs of SAXS/WAXS and PDDF curves from atomic structures and evaluate these computational methods in terms of trade-offs between accuracy and computational efficiency using structures that range in size from 1 kDa molecular weight (MW) with 66 non-hydrogen atoms to 1.1 MDa MW with 81 960 non-hydrogen atoms. The examples demonstrate the use and power of the theoretical PDDFs simulated from atomic structures, for example, distinguishing real PDDF features from artefacts and identifying the origins of PDDF peaks using partial structure modeling.

## 2. Coordinate-based PDDF and XS calculation methods

In this section, we will describe three methods that compute PDDFs and XS directly from the atomic structure of the molecule in solution using implicit solvent. The first is an approximate method based on a point-charge model, in which all the electrons of an atom are assumed to be localized at the center of the atom and behave as a point with charge *Z*. The second method utilizes the real atomic X-ray form factors and the Debye formula. The third is a fast implementation of the second method using distance histograms with a modified Debye formula. All three methods have been implemented in the stand-alone software *SolX 3.0*, which is available at https://12idb.xray.aps.anl.gov/solx.html.

PDDF and XS calculations based on implicit-solvent models can be extended to include components that provide adjustable approximate models for hydration layers and solvent-excluded volumes of the solute molecule (Svergun *et al.*, 1995 ▸; Grishaev *et al.*, 2010 ▸; Schneidman-Duhovny *et al.*, 2010 ▸, 2013 ▸; Poitevin *et al.*, 2011 ▸; Knight & Hub, 2015 ▸; Grudinin *et al.*, 2017 ▸). More accurate methods for calculation of SAXS/WAXS that include solvation-layer atoms have been developed on the basis of explicit-solvent all-atom MD simulations (Chen & Hub, 2014 ▸; Knight & Hub, 2015 ▸; Chatzimagas & Hub, 2022 ▸). However, the computational cost for explicit-solvent all-atom MD simulations makes implementation of these approaches difficult, particularly for large, megadalton and larger, biomolecular assemblies. Furthermore, as described below, explicit solvation-layer atoms can be added to mol­ecular models using implicit bulk solvent approaches to yield computationally efficient algorithms. However, up to now, these algorithms have not been extended to include calculated PDDF patterns based on models. We suggest that the opportunity to combine real-space PDDFs and reciprocal-space XS against experimental data will be a significant advantage to model evaluation and refinement.

### 2.1. Point-charge model for PDDFs and XS

With the point charge (PC) model, the PDDF, *P*(*r*), of a molecular assembly can be approximated by tallying the distances of atom pairs in the assembly:
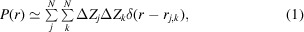
where *r_j,k_* is the distance between the *k*th and *j*th atoms. The symbol δ(…) is the delta function: δ(*x*) = 1 when *x* = 0 and δ(*x*) = 0 when *x* ≠ 0. The term Δ*Z*_*j*_ is the net electron number over the excluded solvent for atom *j*, *i.e.* Δ*Z*_*j*_ = *Z*_*j*_ − ED *V*_*j*_, where *Z_j_* and *V_j_* are the number of electrons and the volume of atom *j*, respectively, and ED is the electron density of the surrounding solvent/buffer. Equation (1) is essentially a distance histogram weighted by the effective electron numbers. This PC model approach provides a quick estimate of the PDDF (PC-PDDF) from the atomic coordinates. Integrating equation (1) will show that the area of the *P*(*r*) function is equal to the square of the total effective electron number of the assembly, *i.e.*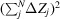.

The XS profile, *I*(*q*), and PDDF, *P*(*r*), are interlinked by the Fourier transform (FT):
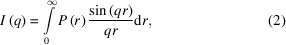
where *q* is the XS momentum transfer, 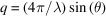 (λ = X-ray wavelength; θ = Bragg angle). *FoXS* calculates the SAXS profile with the same approach, through the FT of the PC-PDDF, which greatly speeds up the SAXS profile simulations (Schneidman-Duhovny *et al.*, 2013 ▸; Förster *et al.*, 2008 ▸). The accuracy of the point charge model PDDF will be verified by more precise PDDF methods in real space or by comparing *I*(*q*) obtained using equation (2) and other direct *I*(*q*) methods in reciprocal space in the later sections. The scattering intensity profile *I*(*q*) can be obtained through numerical integration of the PC-PDDF, as shown in equation (2), and the *I*(*q*) profile converges when the numerical *P*(*r*_*j*_) profile has a distance increment (Δ*r* = *r*_*j*+1_ − *r*_*j*_) of ∼0.1 Å or less. The XS profile calculated through the numerical integration of the PC-PDDF will be denoted as PC-XS. From equation (2), one can infer that the area of *P*(*r*) is equal to *I*(*q* = 0), the forward scattering, which is the square of the total effective electron number (see details in the next section) and closely related to the MW (Orthaber *et al.*, 2000 ▸).

### 2.2. PDDF and XS calculation through the direct Debye formula

Since the PDDF and XS are interlinked by the FT, *P*(*r*) could be calculated through scattering *I*(*q*) as well. As widely applied, the XS of an assembly can be computed using the Debye formula (Svergun *et al.*, 1995 ▸; Zhang *et al.*, 2000 ▸; Zuo *et al.*, 2006 ▸):
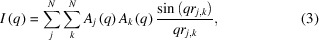
where *A_j_* is the overall scattering form factor of the *j*th atom, and *r_j,k_* is the distance between the *k*th and *j*th atoms. The overall atomic scattering form factor, *A_j_*, can be expressed as follows:
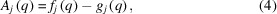
where *f*_*j*_(*q*) is the atomic XS form factor of the *j*th atom or atomic group. Tabulations of atomic form factors are available from quantum chemistry calculations with a finite *q* range up to 24π (∼75) Å^−1^ (Prince, 2004 ▸). These can be fitted with a finite number of Gaussian functions, for example, five Gaussians (5G) in this work. The term *g*_*j*_(*q*) is the dummy-atom form factor of the solvent displaced by atom or atomic group *j*, and it can be approximated in a Gaussian form factor [equation (S3) of the supporting information] (Fraser *et al.*, 1978 ▸; Svergun *et al.*, 1995 ▸; Zhang *et al.*, 2000 ▸). This direct Debye calculation for XS is denoted as DD-XS in this study.

As mentioned above, *I*(*q*) and *P*(*r*) are interlinked and *P*(*r*) can be calculated by the inverse FT of *I*(*q*):
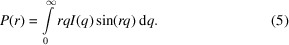
Substituting equation (3) into equation (5), *P*(*r*) can be rewritten as
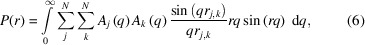
where *r* is a given distance within the molecule or assembly. Since *A*_*j*_(*q*) in equation (4) can be written as a summation of a few Gaussian functions, so can the term *A*_*j*_(*q*)*A*_*k*_(*q*):
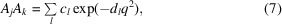
where *c* and *d* are constants. For the summation of Gaussian functions, the integral of equation (6) has an analytical solution, and *P*(*r*) can be written as the summation of a set of distance-weighted Gaussian functions, *i.e.*
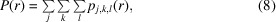
where
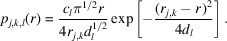
Equation (8) provides an analytical approach to compute the PDDF for a molecular assembly based on the finite atomic form factor *q* ranges. This analytical PDDF calculated from the direct Debye formula is denoted as DD-PDDF. The currently available *q* range for atomic form factors is sufficient to generate an accurate DD-PDDF because further extension of the form factor to higher *q* ranges will only yield additional *p*_*j*,*k*,*l*_(*r*) terms with small *c_l_* values, which are negligible in equation (8).

The computational cost of DD-XS calculation [equation (3)] is proportional to the square of the number of atoms (*N*_A_) and the number of *q* values (*N*_Q_), *i.e.O*(*N*_A_*N*_A_*N*_Q_), and the cost of DD-PDDF calculation [equations (5)–(8)] is proportional to the square of the number of atoms, the number of distance values (*N*_R_) and the square of the number of Gaussians (*N*_G_) in equation (4), *i.e.O*(*N*_A_*N*_A_*N*_R_*N*_G_*N*_G_). It will take a normal desktop computer ∼6–8 min to compute the XS (300 *q* values) or DD-PDDF (∼180 *R* values) using the above approach for bovine serum albumin (BSA), a medium-size protein with ∼4400 non-hydrogen atoms and a molecular weight of 62 kDa. Since the costs of DD-XS and DD-PDDF calculations are both proportional to *N*_A_ squared, these methods will quickly become cumbersome and not practical for molecules larger than 100 kDa: for example, DD-PDDF calculation requires ∼6 h for the 1.3 MDa satellite tobacco mosaic virus (STMV).

### 2.3. Distance-histogram algorithm for fast PDDF and XS calculations

To reduce the computational cost, a pair distance histogram algorithm is employed in XS and PDDF calculations. Instead of computing on every individual atom pair (pair *j, k*) in equation (3), the atom-pair distance histograms are generated before the scattering and PDDF calculations. To maintain the calculation accuracy, we categorize atoms or atomic groups according to their X-ray-related properties, such as X-ray form factor, electron number and volume. In biomolecular assemblies, there are a small number (10–15) of frequently occurring atom or atom-group types (*i.e.* a non-hydrogen atom with attached hydrogen atoms), for example, C, CH, CH_2_, CH_3_, N, NH *etc.* (Svergun *et al.*, 1995 ▸). We generate the distance histograms between atom types, and the scattering and PDDF calculations can be rewritten as equations (9) and (10), respectively:
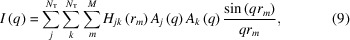

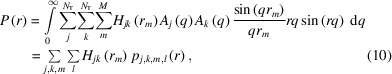
where *H*_*jk*_(*r*_*m*_) is the histogram of atom-pair distances between atom types *j* and *k*. The cost of the distance-histogram-based XS (DH-XS) calculation [equation (9)] is *O*(*N*_T_*N*_*T*_*N*_R_*N*_Q_), and the cost of the distance-histogram-based finite analytical PDDF calculation [DH-PDDF, (10)] is *O*(*N*_T_*N*_T_*N*_R_*N*_G_*N*_G_). *N*_T_ is the number of atom/atom-group types in the molecule. *N*_A_ is often in the range of 10^3^–10^6^, while *N*_T_ is much smaller, typically 10–15 for biomolecules. Therefore, the costs of equations (9) and (10) are dramatically reduced when using distance histograms. The overall costs of distance-histogram-based (both DH-XS and DH-PDDF) calculations are actually dominated by the time spent on generating distance histograms, which is *O*(*N*_A_*N*_A_). The cost of the PC-PDDF calculation [equation (1)] is also *O*(*N*_A_*N*_A_). On average, the computational time drops >100-fold from the DD-PDDF to the DH-PDDF, and approximately fourfold further from the DH-PDDF to the PC-PDDF due the simplicity of the latter. More detailed performance comparisons can be found in Table 1 ▸. In the present study, fast algorithms for PDDF and XS calculations based on implicit solvent have not been included to account for the solvation layer. However, several approaches elsewhere have been developed to do so (Svergun *et al.*, 1995 ▸; Grishaev *et al.*, 2010 ▸; Schneidman-Duhovny *et al.*, 2010 ▸, 2013 ▸; Poitevin *et al.*, 2011 ▸; Knight & Hub, 2015 ▸; Grudinin *et al.*, 2017 ▸). The distance-histogram method is amenable to being extended using these approaches, and the described ways to calculate both PDDF and XS profiles will provide a means to compare models and experiment in both real and reciprocal space.

### 2.4. Comparison of accuracy in PDDF and XS calculations

**Figure 1:**
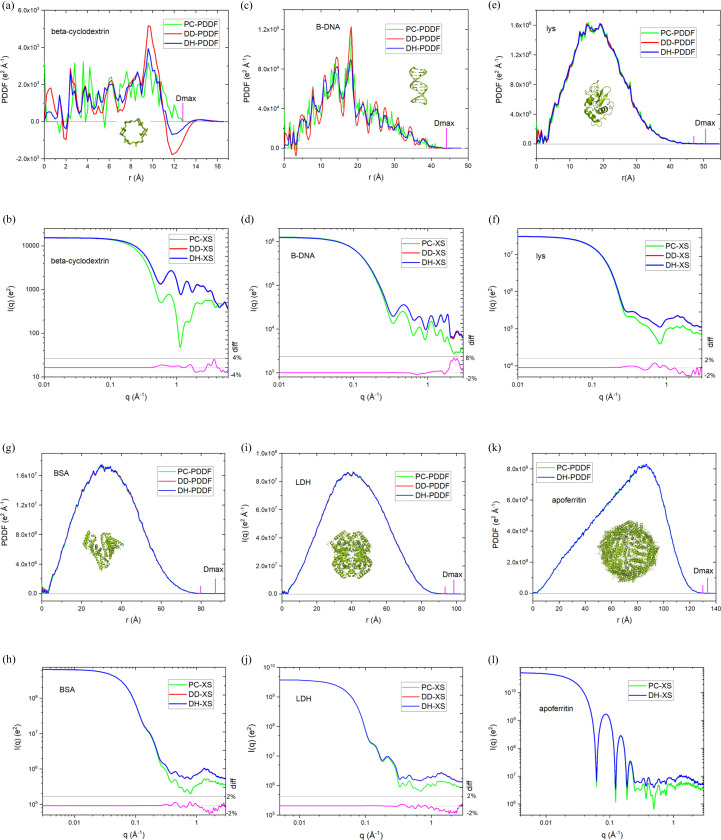
Comparison of PDDF (first and third rows) and XS (second and fourth rows) profiles obtained through different theoretical methods, for molecules of <1 kDa to ∼0.5 MDa. PDB codes of these molecules are listed in Table 1 ▸. In PDDF figures (*e*), (*g*), (*i*) and (*k*), the long vertical magenta line marks the true Dmax, while the short line marks the position of the *P*(*r*) value at ∼0.1% of the peak maximum. The distances between the two marks are 3–7 Å. In (*k*) and (*l*), only the DH-PDDF, PC-PDDF, DH-XS and PC-XS profiles are provided because DD-PDDF and DD-XS profiles with sufficient data points will take too long for the ∼0.5 MDa apo-ferritin. The magenta curves in (*b*), (*d*), (*f*), (*h*) and (*j*) are percentage difference (right *y* axis) between the DH-XS and DD-XS curves, and diff = (DH-XS/DD-XS − 1)100%. In all cases, diff < 0.3% within *q* ≤ 0.30 Å^−1^. The dashed line is the zero line.

The DD-XS [equation (3)] and DD-PDDF [equations (6)–(8)] approaches serve as high-fidelity references for XS and PDDF calculations, respectively, because they employ the fewest approximations. Fig. 1 ▸ displays the simulated PDDF and XS profiles for representative molecular assemblies, ranging from ∼1 kDa to ∼0.5 MDa. In this broad size range of molecular assemblies, the PDDF profiles obtained via the distance-histogram (DH-PDDF) approach are close to those from the DD-PDDF method, but have the advantage of accelerating calculations by >100-fold. The PC-PDDF profiles for small molecular assemblies with relatively low atom density, illustrated in Fig. 1 ▸ with β-cyclo­dextrin (β-CD) and B-form DNA duplex, exhibit fluctuations that differ significantly from those obtained from the DD-PDDF method. However, the difference becomes smaller for larger (BSA, apo-ferritin, *etc.*) or more compact (*e.g.* lysozyme) assemblies. As shown in Figs. 1 ▸(*e*), 1 ▸(*g*), 1 ▸(*i*) and 1 ▸(*k*), the difference between the PC-PDDF and DD-PDDF profiles in the central distance range is smaller than 1–2%. The good agreement between the PC-PDDF and DD-PDDF profiles for proteins with compact folds can be ascribed to the similar electron-density functions for the substituent non-hydrogen atoms (*i.e.* C, N, O *etc.*) and the relatively narrow broadening due to the element electron-density distribution function (Fig. S1 of the supporting information).

The distance-histogram approach also exhibits a high fidelity in XS calculations, as demonstrated in Figs. 1 ▸(*b*), 1 ▸(*d*), 1 ▸(*f*) and 1 ▸(*h*). For example, using distance histograms with a distance increment of 0.2 Å, the scattering intensity difference between the DH-XS and DD-XS methods is <0.1% within *q* < 0.2 Å^−1^ and <2% in the *q* range of 0.2–3.0 Å^−1^ for BSA. However, the computational time is decreased ∼200-fold for BSA. Throughout the wide range of assemblies, XS profiles obtained through the FT of the PC-PDDF (PC-XS) are a close match to the DD-XS profiles at small angles (*q* < ∼0.1–0.2 Å^−1^); in the high-angle range (*q* > 0.2 Å^−1^), the scattering oscillation features of the PC-XS profiles generally resemble those of the DD-XS or DH-XS profiles, but the intensities deviate. *FoXS* adopts an empirical adjustment of equation (2) to amend the inaccuracy in the high-angle intensities (Förster *et al.*, 2008 ▸; Schneidman-Duhovny *et al.*, 2013 ▸). Among these computational approaches, considering both the computational speed and accuracy, we find that the distance-histogram method is the most efficient for both XS and PDDF simulations.

## 3. Applications of coordinate-based PDDF calculation

### 3.1. Verification tool for IFT-calculated PDDFs

**Figure 2:**
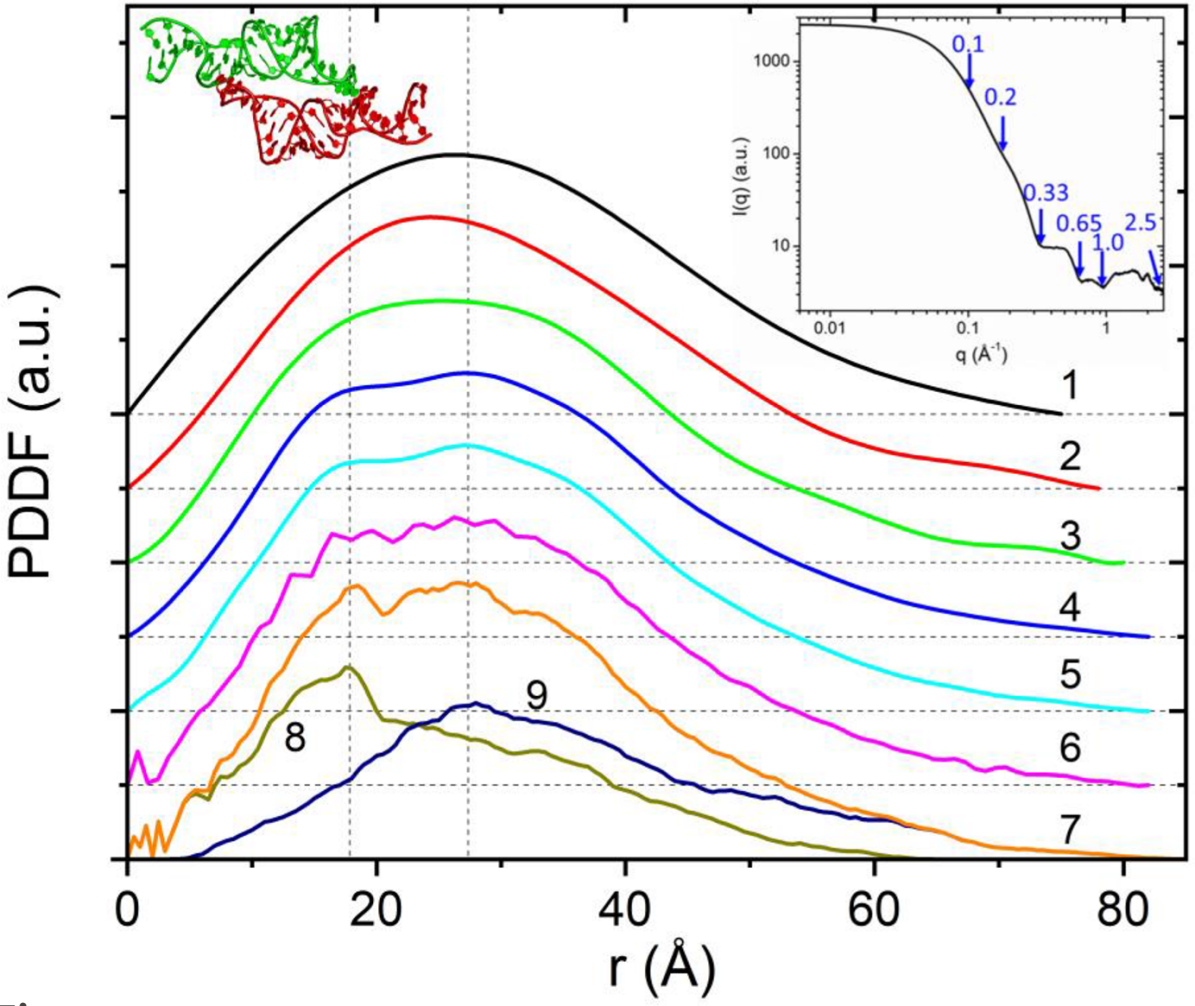
Experimental and simulated total and partial PDDFs for dimeric tetra-loop receptor RNA (tectoRNA). Right upper inset: experimental XS data [reused from Zuo *et al.* (2008 ▸)] of tectoRNA, with a *q* range of 0.006–2.5 Å^−1^. Left upper inset: tectoRNA (PDB code 2jyf; Zuo *et al.*, 2008 ▸) in a cartoon model, generated using *PyMOL* (https://www.pymol.org/). The two monomeric units are displayed in green and red. Curves 1–6 show the variation in PDDF profiles obtained using *GNOM* and adjusting the maximal *q* value (qmax) for the input data: 1, qmax = 0.10; 2, qmax = 0.20; 3, qmax = 0.33; 4, qmax = 0.65; 5, qmax = 1.00; and 6, qmax = 2.50. PDDF curves 7 and 8 are the DD-PDDF profiles directly calculated from the tectoRNA dimer and monomer coordinates, respectively. Curve 9 is the *P*^corr^(*r*) between the two monomeric units. All the PDDFs were plotted on the same scale, but the experimental PDDFs were vertically offset for clarity. The black thin dashed lines are zero lines.

The PDDF calculated from SAXS data using the direct or indirect FT (Svergun, 1992 ▸; Glatter, 1977 ▸) could introduce artefacts, such as false oscillations or loss of fine features due to over-smoothing or working beyond the software limits, and therefore may lead to incorrect interpretation. For example, the program *GNOM* (Svergun, 1992 ▸) is one of the most popular and powerful experimental PDDF analysis software tools, and its default regulation parameters are optimized for globular proteins and SAXS data. Working beyond these software limits due to lack of choices and/or unintentional misuse could produce artefacts. PDDFs calculated from model structures using the methods described above can help discriminate between structure-based features and computational artefacts caused by the IFT or limited XS data range. Fig. 2 ▸ displays experimental and theoretical PDDFs of a tectoRNA molecule, an RNA symmetric homo-dimer (Zuo *et al.*, 2008 ▸). The experimental PDDF profiles (1–6) were obtained from the IFT of partial or complete experimental XS data sets using *GNOM* (Svergun, 1992 ▸). The features of the resulting PDDF profiles, *e.g.* the peak shape and Dmax value, moderately depend on the input data range. In particular, the double peaks at 18 and 24 Å in profiles 4 and 5 were not obvious in other PDDF profiles that utilized smaller or larger data ranges. Profile 6 utilizes the full range of scattering data up to 2.5 Å^−1^, which goes far beyond the normal SAXS region, and exhibits ripples on the top of the main peak. The theoretical PDDF simulations confirm these double peaks and ascribe the short-distance peak to the PDDF of individual RNA units and the long-distance peak to the inter-unit distance correlations. This example demonstrates the possible ambiguity in a PDDF from the FT of the XS when using improper data ranges or working beyond software limits. Some of the data processing in Fig. 2 ▸ is intentional misuse of *GNOM* – for example, input data with too narrow or too wide *q* ranges – but these cases reflect some realities such as inadequate experimental data ranges, lack of choice of software and attempts to extract structural information that WAXS data could provide. Theoretical PDDF simulations could provide guidance for such situations.

### 3.2. Resolving conflicting models for molecules in solution state

**Figure 3:**
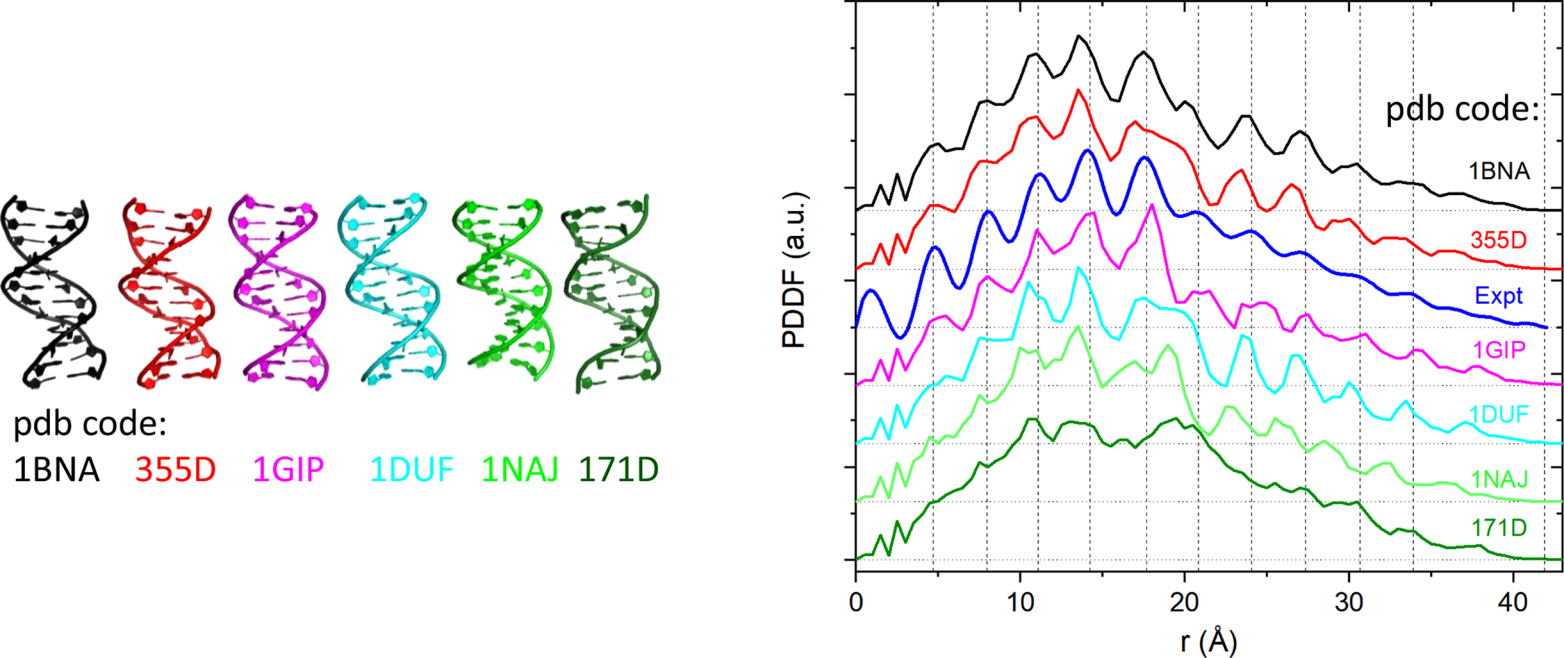
Experimental PDDF and DH-PDDF profiles for Drew–Dickerson DNA. The blue profile is the PDDF calculated for experimental XS data using *GNOM* (Fig. S2). The other PDDFs were calculated from labeled PDB structures using the DH-PDDF approach. PDB codes 1bna and 355d (Shui *et al.*, 1998 ▸) are crystallographic structures, and PDB codes 1gip, 1duf (Tjandra *et al.*, 2000 ▸), 1naj (Wu *et al.*, 2003 ▸) and 171d are NMR structures. PDDFs were vertically offset for clarity. The horizontal dotted lines are respective zero baselines. The vertical dashed lines label the experimental PDDF peaks and Dmax.

The biomolecular configuration under physiological or solution conditions is more relevant to biological function. However, high-resolution structure measurements are often performed far away from such conditions, for instance, in the crystalline state for crystallography and in a frozen state for cryo-EM, therefore causing possible distortion from the free solution phase structure (Hura *et al.*, 2019 ▸; Zuo & Tiede, 2005 ▸). Another source of structural discrepancy could arise from the limitations of structural determination techniques. For example, the solution NMR technique tends to lack sufficient long-distance restraints (Grishaev *et al.*, 2005 ▸; Zuo *et al.*, 2008 ▸). Despite being a relatively low resolution structural technique, solution XS can be used to resolve conflicting models for molecules in a solution state. Fig. 3 ▸ is a revisited case: Drew–Dickerson DNA. The previous study demonstrated that solution XS can identify a preferred model from a variety of published crystallographic and NMR structures describing the solution-state structure (Zuo & Tiede, 2005 ▸). However, evaluation of these models from the PDDF perspective reveals additional structural insights. For example, the experimental PDDF exhibits an oscillatory pattern arising from the layered ladder structure of duplex DNA (Fig. S2). Specific features of the PDDF profile reflect the degree of regularity of the repeated DNA structure. Among the surveyed models, PDB structure 1gip (Kuszewski *et al.*, 2001 ▸) matches the experimental PDDF best in terms of the alignments of PDDF peak positions, followed by PDB structure 1bna (Drew *et al.*, 1981 ▸). PDB structure 171d (Schweitzer *et al.*, 1994 ▸) exhibits a poor structural regularity and fits the least well to the experimental data. The peaks of structure 1bna are slightly shifted towards lower distance values than those of structure 1gip, reflecting the shorter base rise in structure 1bna. Structure 1gip was refined from PDB structure 1dup (Cedergren-Zeppezauer *et al.*, 1992 ▸) using different base–base potential interaction, which results in the increase in base rise and closer agreement to experiment.

### 3.3. Identification of the origin of PDDF features

Although the PDDF is a real-space function, detailed interpretation of PDDF profiles is complicated by the pair-distance representation of molecular structure, weighted by atomic scattering factors. PDDF simulation from selected atomic groups and molecular substructures can help identify and understand the origin of PDDF features. For example, for a system consisting of two subunits, A and B, the PDDF can be written as
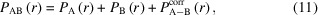
where the first two terms are the PDDFs of the individual subunits and the last term is the inter-subunit distance correlation function. For a system with *N* (>2) subunits, its PDDF can be dissected into the contributions of single subunits and the correlations between subunit pairs:
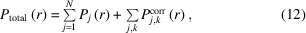
where *P*_*j*_(*r*) is the PDDF for subunit *j*. 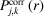 is the correlation between subunits *j* and *k* and can be calculated from equation (11). Simulations of these partial structures can be very helpful in understanding the origins of the PDDF features.

**Figure 4:**
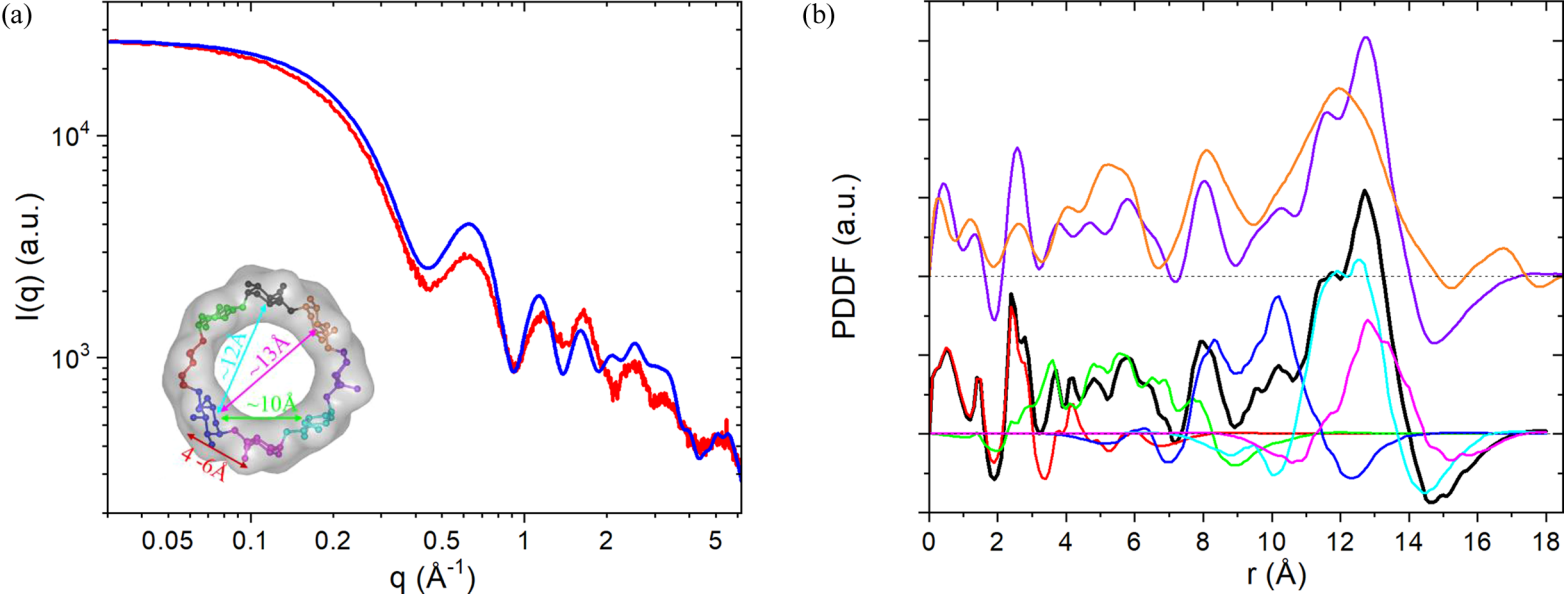
Experimental and simulated XS and PDDF profiles for γ-CD. (*a*) Superimposed simulated (blue) and experimental (red) XS profiles for γ-CD in water. Inset: the atomic model (PDB code 1d3c; Uitdehaag *et al.*, 1999 ▸) of γ-CD used in the calculation. The eight glucose units of γ-CD are coded in different colors. (*b*) Whole and partial PDDFs for γ-CD. The upper PDDF curves were calculated from the full scattering profiles in (*a*) using *GNOM*: orange, from experimental data; and purple, from simulated data. The lower profiles were directly simulated from the atomic structure using the DD-PDDF approach: red, for all single glucose units; green, inter-unit distance correlations (*P*^corr^) for all nearest neighboring unit pairs; blue, inter-unit distance correlations for all unit pairs separated by one unit; cyan, inter-unit distance correlations for all unit pairs separated by two units; magenta, inter-unit distance correlations for all unit pairs separated by three units; and black, whole PDDF for γ-CD. The black curve equals the summation of red, green, blue, cyan and magenta. The negative values at long distances for both the whole and partial PDDFs arise from the negative net electron number of the CH_2_ group in water. The black and purple curves are very close if superimposing the (dashed) baselines. The peak at 17 Å in the experimental PDDF (orange) could arise from the solvation layer. The simulated PDDFs reproduced most of the features in the experimental one, and the partial structure simulations help identify their origins. XS data were collected at beamlines 12-ID-B and 12-ID-C of the Advanced Photon Source at Argonne National Laboratory.

Fig. 4 ▸ shows the PDDF analyses for γ-CD. The PDDF derived from the experimental data consists of a few peaks up to 18 Å, and the simulated PDDFs from the atomic structure successfully reproduce most of the experimental PDDF peaks. The simulations on the partial structures (*i.e.* glucose subunits) show that PDDF peaks within 3 Å arise from the internal structure of glucose subunits, while those in the range of 3–15 Å can be ascribed to various inter-unit correlations. The peak at ∼17 Å in the experimental PDDF but absent in the simulated γ-CD-only PDDFs could arise from the solvation layer. A water shell around the outer surface of γ-CD can reproduce the PDDF peak at ∼17 Å, significantly improving the agreement between experimental and simulated XS profiles in the low-*q* region (Fig. S3). This example demonstrates that PDDF simulations together with atomic structure manipulations can help understand the origins of PDDF features and identify problems in data misfits, which are often challenging using reciprocal-space XS data alone.

### 3.4. Some general features of PDDFs

One of the important parameters obtained from the PDDF profile is Dmax, the largest dimension of the molecular assembly under study. In a PDDF, Dmax is the shortest distance where the PDDF probability is zero at this distance and beyond, *i.e.P*(*r* ≥ Dmax) = 0. Simulations of PDDFs show that they often have a long tail even for globularly well folded molecules because of the fuzzy molecular surfaces, which makes it difficult to determine the true Dmax unambiguously. Therefore, there is a tendency to underestimate Dmax values. On the other hand, the true Dmax could be represented by only a very small number of pairs in some cases. For example, in Figs. 1 ▸(*e*), 1 ▸(*g*), 1 ▸(*i*) and 1 ▸(*k*) at the positions where the *P*(*r*) value is 0.1% of the maximum PDDF peak (marked by the short vertical lines), *P*(*r*) could visually be considered close enough to be zero. The apparent Dmax estimated from the short marker position is 3–7 Å shorter than the true Dmax in these selected molecules. The gap could be larger for more extended molecules. Therefore, it is a question of whether the true Dmax value is meaningful or experimentally approachable and whether there is a need to define a more experimentally meaningful Dmax. Theoretical PDDFs could be used for such studies. The solvation layer, which is not included in current simulations, would increase the apparent Dmax and could complicate the measurement of the true molecular Dmax if it introduces additional features, such as the long-distance PDDF peak observed for the aqueous γ-CD sample discussed in Section 3.3.

**Figure 5:**
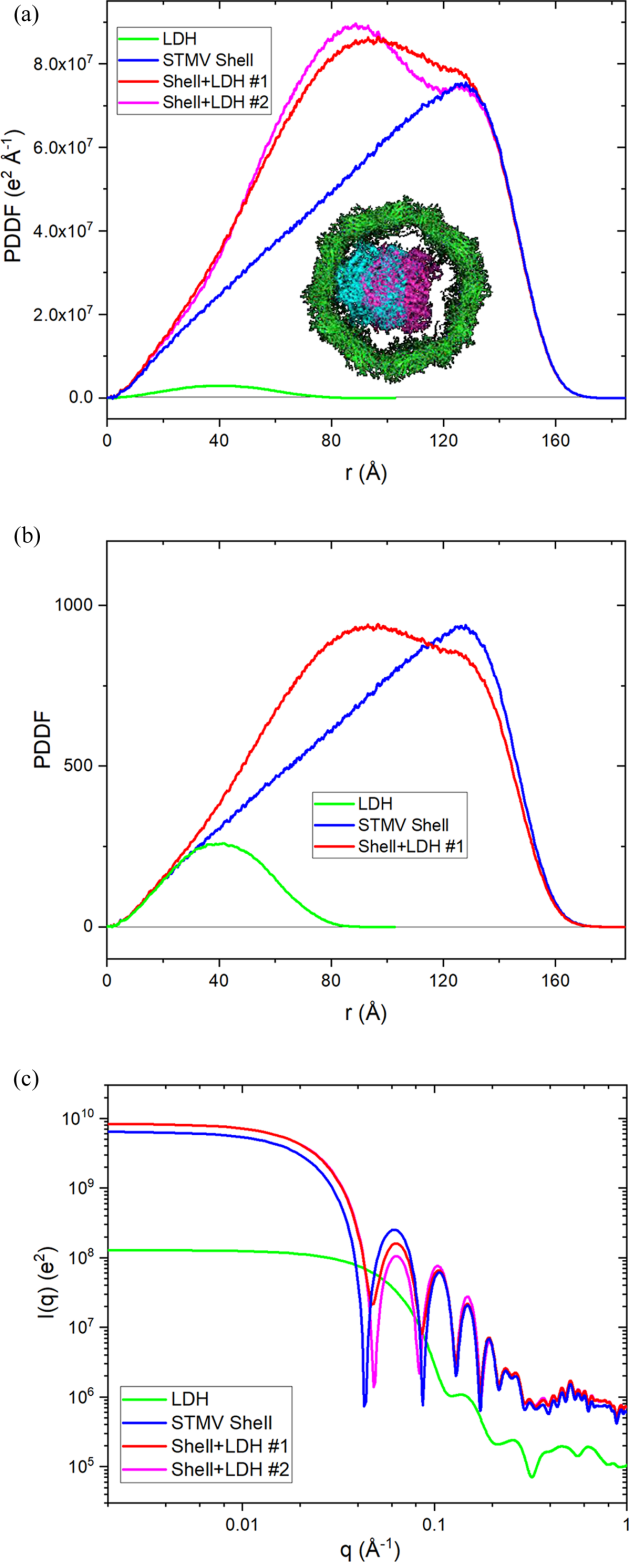
Simulated PDDFs and scattering profiles for the artificial 1:1 adduct of STMV [green structure in the inset of (*a*), PDB code 1a34; Larson *et al.*, 1998 ▸] protein shell and LDH (cyan/magenta structure, PDB code 1i10; Read *et al.*, 2001 ▸) using distance-histogram methods. PDDF profiles are shown in (*a*) and (*b*): blue, protein shell alone; green, RNA alone; and red, protein shell + RNA. (*a*) PDDFs for individual molecules. The ratios between PDDF profiles in this presentation are the same as the PDDFs derived from SAXS data normalized by respective molar concentrations. The inset of (*a*) is the cross-section view of two 1:1 STMV–LDH adducts, differentiated by the LDH position in the STMV shell. Red and magenta PDDF profiles are for the two STMV–LDH adducts: green shell + cyan LDH and green shell + magenta LDH, respectively. (*b*) PDDFs in (*a*) rescaled to have the same ratios as the PDDFs derived from SAXS data normalized by respective mass concentrations. (*c*) Simulated XS profiles.

Another frequently encountered problem is the normalization of PDDFs when comparing closely related assemblies, *e.g.* comparing mass distribution before and after multicomponent assembly. Proper normalization of PDDFs will be critical for such data interpretation. As discussed in previous sections, the area under theoretical PDDFs is equal to the square of the number of effective electrons in the assembly. When dealing with experimental data, normalization on *I*(*q*) and the PDDF could be tricky, and improper normalization would lead to wrong interpretation. For example, Fig. 5 ▸(*a*) displays PDDFs of the STMV protein shell, l-lactate de­hydrogenase (LDH) and two hypothetical adducts made by inserting a single LDH molecule inside a host STMV shell. The two adducts differ by the position of LDH within the STMV shell, with one positioned close to the shell wall [cyan trace, Fig. 5 ▸(*a*) inset] and the other having the guest LDH placed at the capsid center [magenta trace, Fig. 5 ▸(*a*) inset]. The differences in the shapes of the PDDFs for the two STMV–LDH assemblies can be intuitively understood from the corresponding structures, and the difference areas between the two adducts can be seen to be constant, a consequence of the size and stoichiometry of the LDH guest in the adduct assembly.

Such intuitive inferences could not be understood from inspection of SAXS patterns alone. In analysis of unknown structures of multicomponent assemblies by SAXS and IFT-PDDF approaches, SAXS data are often normalized, since this provides the most straightforward way to characterize the difference in reciprocal space. However, the FT/IFT of incorrectly normalized SAXS can be shown to generate PDDF patterns which may lead to incorrect interpretation. When comparing PDDFs between molecules, they should all be calculated on the single molecular particle level, which is the default setting for coordinate-based PDDF calculation. Therefore, IFT-PDDFs should be obtained from SAXS data normalized by sample molar concentration (mol l^−1^). However, in SAXS practice, mass concentration (mg ml^−1^) is favored over molar concentration; therefore, SAXS data have a high chance of mistakenly being normalized by mass concentration for IFT-PDDF comparison, which would lead to the wrong conclusion. For example, Fig. 5 ▸(*b*) shows the PDDF curves from Fig. 5 ▸(*a*), but normalized by MW, which is equivalent to IFT-PDDFs of SAXS data normalized to mass concentration. Fig. 5 ▸(*b*) easily provides an impression that LDH incorporation causes mass rearrangements at long distances, possibly in the protein shell. These considerations demonstrate the utility of developing model-based PDDF simulation approaches to complement SAXS and IFT analyses.

## 4. Concluding remarks

Coordinate-based SAXS/WAXS simulation has been widely used for scattering data analysis, while direct coordinate-based PDDF simulation has not been fully exploited. As a real-space function, the PDDF can provide a complementary, more intuitive, view for interpreting XS data. Here, we presented theoretical methods for direct PDDF simulation from atomic coordinates. The theoretical PDDF profiles free of artefacts can be used as a guide to check artificial features in IFT-calculated PDDFs and to interpret experimental X-ray data on the basis of real-space PDDF features. Advances in biological and materials science research include increases in both the dimensional scale and complexity of natural and synthetic systems that can be investigated using XS. The fast simulation algorithms described here make model-based PDDF investigation a practical approach for study of these new materials. Key structural parameters can be derived from PDDFs, such as Dmax, molecular conformation, shape and low-resolution *ab initio* models. However, in general, the PDDF is still under-exploited. In the emerging area of stimulus-responsive nanoscale and biological smart materials, many function through conformational changes or molecular recognition. Studies on such relative structural changes could benefit from the PDDF approach and real-space constituent and partial structure analyses.

## 5. Related literature

The following reference is only cited in the supporting information for this article: Li *et al.* (2019 ▸).

## Supplementary Material

Supporting information. DOI: 10.1107/S1600576724007222/uz5012sup1.pdf

## Figures and Tables

**Table 1:** Computational costs for a few molecules in this study All computation was carried out on a desktop computer with Core i7-8700 CPU @ 3.20 GHz and 16 GB RAM. DD/DH/PC methods for calculating the XS and PDDF have been implemented in *SolX 3.0*, which can be downloaded from https://12idb.xray.aps.anl.gov/solx.html.

Name	PDB code	MW (kDa)	Number of non-hydrogen atoms	Dmax	DD-XS with 300 *q* points (s)	DD-PDDF (s)†	DH-XS/DH-PDDF (s)‡	PC-XS/PC-PDDF (s)†§
β-CD	1btc (Mikami *et al.*, 1993 ▸)	0.97	66	12.6	<0.1	<0.1	<0.1	<0.1
B-DNA	1gip	7.3	486	44.1	5	13	<0.1	<0.1
Lysozyme	2lyz (Diamond, 1974 ▸)	14.2	1001	50.6	19	24	<0.1	<0.1
BSA	3v03 (Majorek *et al.*, 2012 ▸)	62.1	4385	87.2	362	449	2	1
LDH	1i10	145.2	10272	98.6	1192	2482	10	3
Apo-ferritin	6z6u (Yip *et al.*, 2020 ▸)	497.2	35304	133.6	23682	29170	101	26
STMV	1a34	1174.1	81960	181.5	167262	202181	782	190

† The distance increment was set as 0.5 Å.

‡ The cost of DH-XS and DH-PDDF calculation is roughly the same because building the distance histograms is the rate-determining step for both.

§ The cost of PC-XS and PC-PDDF calculation is roughly the same because the cost of numerical computation of equation (2) is negligible.
