# Supplementary material for: Coordinate-based simulation of pair distance distribution functions for small and large molecular assemblies: implementation and applications
Source: J Appl Crystallogr. 2024 Sep 17;57(Pt 5):1446–55. doi: 10.1107/S1600576724007222 (PMC11460383; doi:10.1107/S1600576724007222)
Supplement: Supplementary file 1 [file j-57-01446-sup1.pdf]

JOURNAL OF
APPLIED
CRYSTALLOGRAPHY

Volume 57 (2024)

Supporting information for article:

Coordinate-based simulation of pair distance distribution functions for small and large molecular assemblies: implementation and applications

Xiaobing Zuo and David M. Tiede

S1. Theory on Coordinate Based X-ray Scattering Calculation

The X-ray scattering of an assembly can be computed using Debye formula (Eq. S1) (Svergun *et al.*, 1995; Zhang *et al.*, 2000; Zuo *et al.*, 2006).

$$I(q) = \sum_{j}^{N}\sum_{k}^{N} A_j(q)A_k(q)\frac{sin(qr_{j,k})}{qr_{j,k}} \tag{S1}$$

where A_j is the overall scattering form factor of the *j*th atom, $r_{j,k}$ is the distance between *k*th and *j*th atoms, and q is the X-ray scattering transfer momentum. The overall atomic scattering form factor A_j, can be expressed as follows:

$$A_j(q) = f_j(q) - g_j(q) \tag{S2}$$

where $f_j(q)$ is the atomic X-ray scattering form factor of the *jth* atom or atomic group. Atomic form factors can be fit with a finite number of Gaussian functions, for examples, five Gaussians (5G) in this work. $g_j(q)$ is the dummy atom form factor of the solvent displaced by atom or atomic group j, and a Gaussian form factor is used (Eq. 3).(Fraser *et al.*, 1978; Svergun *et al.*, 1995; Zhang *et al.*, 2000)

$$g_j(q) = \rho_S Rom^3 V_j e^{-q^2 Rom^2 V_j^{2/3}/4\pi} \tag{S3}$$

where V_j is the volume of the *j*th atom or atomic group, and ρ_s is the X-ray scattering length density (xSLD) of the solvent or buffer.

S2. PDDF calculation through Debye X-ray scattering formula

X-ray scattering, I(q), and pair distance distribution function, P(r), are interlinked and P(r) can be calculated by the inverse Fourier transform of I(q) (Eq S1),

$$P(r) = \int_0^\infty rqI(q)\sin(rq)\,dq \tag{S4}$$

Substituting Eq S1 into Eq S5, *P(r)* can be rewritten as as

$$P(r) = \int_0^\infty \sum_{j}^{N}\sum_{k}^{N} A_j(q)A_k(q)\frac{sin(qr_{j,k})}{qr_{j,k}} rq\sin(rq)\,dq \tag{S5}$$

where r is the given distance within the molecule or assembly. Using the 5G format atomic form factors, A_j in Eq S2 and A_jA_k in Eq S1 become a summation of a series of Gaussian functions:

$$A_j = \sum_m c_{jm} e^{-d_{jm}q^2} \tag{S6}$$

$$A_jA_k = (\sum_m c_{jm} e^{-d_{jm}q^2})(\sum_n c_{kn} e^{-d_{kn}q^2}) = \sum_{l=m+n} c_l e^{-d_l q^2} \tag{S7}$$

where c and d are constants.

Substituting Eq S7 into Eq S5, the pair distance distribution function, P(r), becomes:

$$\begin{aligned} P(r) &= \sum_{j,k}\sum_l \int_0^\infty rqc_l e^{-d_l q^2} \times \frac{sin(r_{jk}q)}{r_{jk}q} \times sin(rq)dq \\ &= \sum_{j,k}\sum_l p_{j,k,l}(r) \end{aligned} \tag{S8}$$

The above integral has an analytical expression,

$$\begin{aligned} p_{j,k,l}(r) &= \int_0^\infty rqc_l e^{-d_l q^2} \times \frac{sin(r_{jk}q)}{r_{jk}q} \times sin(rq)dq \\ &= \frac{c_l \pi^{\frac{1}{2}} r}{4 r_{j,k} d_l^{\frac{1}{2}}} \left(e^{-\frac{(r_{j,k}-r)^2}{4d_l}} - e^{-\frac{(r_{j,k}+r)^2}{4d_l}} \right) \end{aligned} \tag{S9}$$

The second term can be ignored because it only contributes significantly when r is negative. Therefore, Eq S9 becomes:

$$p_{j,k,l}(r) = \frac{c_l \pi^{\frac{1}{2}} r}{4 r_{j,k} d_l^{\frac{1}{2}}} e^{-\frac{(r_{j,k}-r)^2}{4d_l}} \tag{S10}$$

When j=k, r_{jk}=0 and the above formula can be further simplified:

$$p_{j,k,l}(r) = \int_0^\infty rqc_l e^{-d_l q^2} \times sin(rq)dq = \frac{c_l \pi^{1/2} r^2}{4 r_{j,k} d_l^{3/2}} e^{-\frac{r^2}{4d_l}} \tag{S11}$$

Atomic form factors can be fit with multiple Gaussians plus one constant (nG+1), for example in 4G+1 or 5G+1 form. Eq S5 also has analytical formula in such cases. Now, Eqs S6 & S7 will have an additional constant term, saying C_0.

$$p_{j,k,0}(r) = \int_0^\infty rqC_0 \times \frac{sin(r_{jk}q)}{r_{jk}q} \times sin(rq)dq = \frac{\pi C_0\, r\, \delta(r - r_{j,k})}{2 r_{j,k}} \tag{S12}$$

where $\delta(...)$ is the delta function. $p_{j,k,0}(r) = 0$ where $r \neq r_{jk}$ and $p_{j,k,0}(r) \approx \frac{\pi C_0}{2\Delta r}$ where $r = r_{jk}$ and Δr is the increment of distance parameter r.

S3. The "additive" property of PDDF for systems with multiple subunits

S3.1. A system with two subunits: B and C

The X-ray scattering amplitude (M(q)) for subunit B can be written as

$$M_B\ (q) = \sum_{jth\ atom\ \ in\ B} A_j(q)e^{iqr_j} \tag{S13}$$

where $A_j(q)$ is the apparent scattering amplitude defined in main text and r_j is the position of jth atom in subunit B. Similar to Eq. S1, the X-ray scattering from subunit B can be written as

$$I_B\ (q) = \left|M_B\ (q)\right|^2 \tag{S14}$$

The total X-ray scattering for the system BC will be:

$$I_{BC}(q) = \left|M_B\ (q) + M_C\ (q)\right|^2 \tag{S15}$$

$$I_{BC}(q) = I_B\ (q) + I_c\ (q) + 2M_B\ (q) * M_C\ (q) \tag{S16}$$

According to Eqs S4, S5 and S16, the PDDF for the system BC will be:

$$P_{BC}(r) = P_B(r) + P_C(r) + P_{B-C}^{corr}(r) \tag{S17}$$

where $P_{B,C}(r)$ is the PDDF for individual subunit B or C and $P_{B-C}^{corr}(r)$ is the distance correlation function between subunits B and C:

$$P_{B,C}(r) = \int_0^\infty rqI_{B,C}(q)\sin(rq)\,dq \tag{S18}$$

$$P_{B-C}^{corr}(r) = \int_0^\infty 2rq(M_B\ (q) * M_C\ (q))\sin(rq)\,dq \tag{S19}$$

S3.2. A system with *N* (>2) subunits

Similar to Eq S14, the total X-ray scattering of this *N*-subunit system will be;

$$I_{total}(q) = \left|\sum_{j=1}^{N} M_j\ (q)\right|^2 \tag{S20}$$

It can be rewritten as below:

$$I_{total}(q) = \sum_{j<k}^{N} \left|M_j\ (q) + M_k\ (q)\right|^2 - \sum_{j=1}^{N} \left|M_j\ (q)\right|^2 \tag{S21}$$

$$I_{total}(q) = \sum_{j<k}^{N} I_{jk}(q) - \sum_{j=1}^{N} I_j\ (q) \tag{S22}$$

where $I_j(q)$ is the X-ray scattering contribution from the subunit *j* and $I_{jk}(q)$ is the X-ray scattering contribution from dual subunit pair *j&k*.

Therefore, the pair distance distribution function of this *N*-subunit system will be

$$P_{total}(r) = \sum_{j<k}^{N} P_{jk}(r) - \sum_{j=1}^{N} P_j\ (r) \tag{S23}$$

where $P_j(r)$ is the PDDF for subunit *j* and $P_{jk}(r)$ is for the sub-system comprising of subunit *j* and *k*.

Similarly, Eq S20 can be also expended as:

$$I_{total}(q) = \sum_{j=1}^{N} \left|M_j\ (q)\right|^2 + \sum_{j<k}^{N} 2M_j\ (q) * M_k\ (q) \tag{S24}$$

and the pair distance distribution function of this *N*-subunit system can be written as:

$$P_{total}(r) = \sum_{j=1}^{N} P_j(r) + \sum_{j,k} P_{j,k}^{corr}(r) \tag{S25}$$

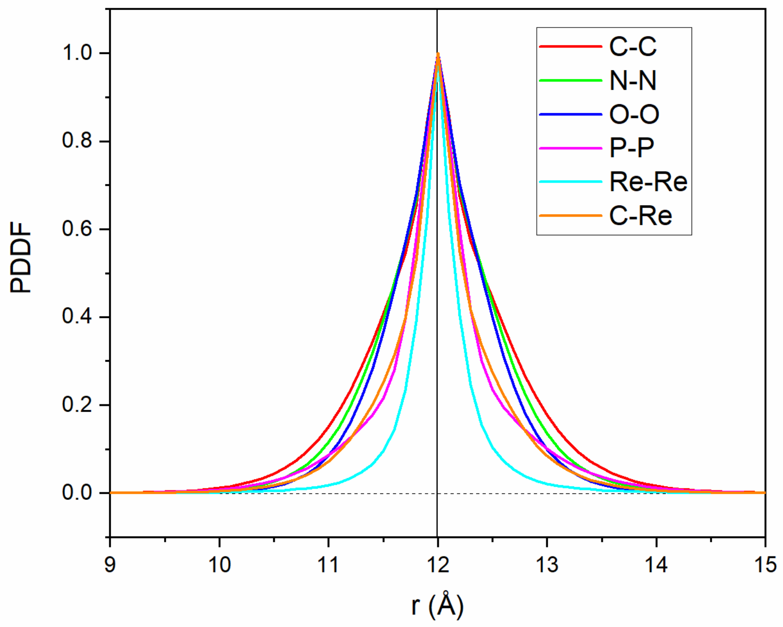

Figure S1 The normalized DD-PDDF for single atom-atom pairs. All atomic pair distances were set at 12 Å. The FWHM (full width at half maximum) of the single atom-pair PDDF peak is within 2 Å. The width is smaller for the atom-pair with heavier atoms.

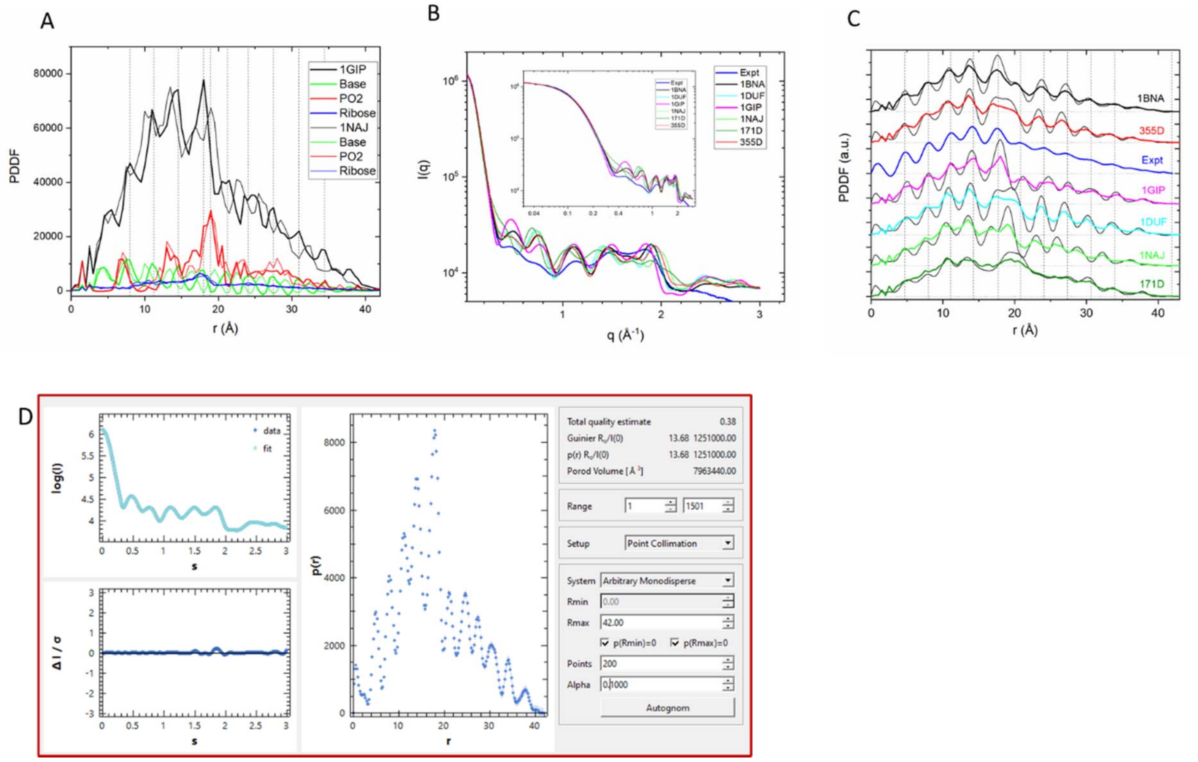

Figure S2 Comparison of PDDF and SAXS/WAXS patterns calculated from crystallographic and NMR coordinates for the Drew-Dickerson B-DNA fragment. (A) DH-PDDFs for structure 1GIP (thick lines) and 1NAJ (thin lines), and its partial structures, base-nucleobases only, PO2-phosphate groups only, ribose-deoxyribose groups only. The PDDF peak positions of the intact DNA molecule align well with those from the base-nucleobases. The mis-match in oscillatory PDDFs of 1GIP & 1NAJ reflects the configurational difference in their repeated layer architecture. The oscillatory patterns of the PDDF profiles are clearly a reflection of the variable parameters of the real-space DNA structure, such as base rise, tilt, twist, etc. The base rise parameters (average ± standard deviation, in Å) for these five pdbs: 1BNA, 3.37±0.17; 355D, 3.29±0.20; 1GIP, 3.43±0.13; 1DUF, 3.36±0.24; 1NAJ, 3.17±0.16; 171D, 3.41±0.49. The amplitude of the base rise standard deviation reflects the degree of the DNA structural regularity. (B). Superimposition of simulated and experimental X-ray scattering profiles in logX-logY(inset) and linearX-logY presentations. (C). Thick profiles are DH-PDDFs. Profiles in thin black lines are PDDFs obtained using GNOM and wide q-range XS shown in (B). There are noticeable differences in respective DH-and GNOM-PDDFs, however, most peak positions still align well in DH-&GNOM PDDFs. (D). Snapshot of GNOM processing of 1GIP simulated XS. For all GNOM PDDFs in (C) including experimental data, the full XS q-range was used and Alpha was set to 0.1. The base rise analyses were performed using the Web 3DNA 2.0(Li *et al.*, 2019) at http://web.x3dna.org.

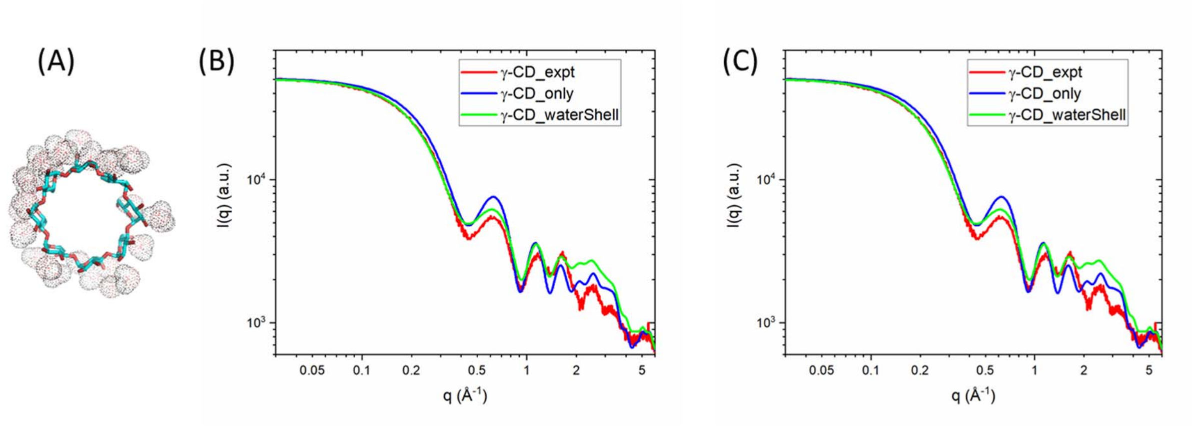

Figure S3 Superimposition of experimental and simulated X-ray scattering and PDDF for γ-cyclodextrin (γ-CD) with and without solvation layer. (A). The structural model of γ-CD (in stick mode) with a water shell solvation layer (in dot mode). The water shell was created from a TIP3 water molecular box generated by charm-gui(https://charmm-gui.org), with a thickness of 3.5 Å around the outer surface of the γ-CD. (B). Experimental (red) data and simulated X-ray scattering profiles of γ-CD with (green) and without (blue) the solvation layer. (C). Experimental (red) and simulated PDDF profiles of γ-CD with (green) and without (blue) the solvation layer. In the PDDF profile, the solvation layer gives rise the peak in the distance range of 16-19 Å. The solvation layer also makes the simulated X-ray scattering profile match better to the experimental data, particularly in the low q region, saying q<0.5 $Å^{-1}$. Those indicate the solvation layer contributes significantly to the experimental X-ray scattering signal of aqueous γ-CD sample.

References

Fraser, R. D. B., MacRae, T. P. & Suzuki, E. (1978). *J. Appl. Cryst.* **11**, 693-694.
Li, S. X., Olson, W. K. & Lu, X. J. (2019). *Nucleic Acids Res* **47**, W26-W34.
Svergun, D., Barberato, C. & Koch, M. H. J. (1995). *J. Appl. Cryst.* **28**, 768-773.
Zhang, R., Thiyagarajan, P. & Tiede, D. M. (2000). *J. Appl. Cryst.* **33**, 565-568.
Zuo, X., Cui, G., Merz, K. M., Jr., Zhang, L., Lewis, F. D. & Tiede, D. M. (2006). *Proc. Natl. Acad. Sci. USA* **103**, 3534-3539.
